# Factors associated with history of suicide attempt among patients with major depressive disorder at a community mental health center in the Peruvian Andes: a case-control study

**DOI:** 10.3389/fpsyt.2026.1915734

**Published:** 2026-08-17

**Authors:** Miguel A. Arce-Huamani, Shiomara C. Pachas-Velapatiño, Cristina N. García-Borjas

**Affiliations:** 1 Programa académico de Medicina Humana, Facultad de Ciencias de la Salud, Universidad Privada Norbert Wiener, Lima, Peru; 2 Universidad Privada San Juan Bautista, Facultad de Ciencias de la Salud, Lima, Peru

**Keywords:** alcohol dependence, borderline personality disorder, major depressive disorder, SDG 3: good health and well-being, suicide attempt

## Abstract

**Background:**

A history of suicide attempt among patients with major depressive disorder has been associated with clinical, psychosocial, and familial vulnerabilities, yet evidence from community mental health services in underserved Andean settings remains limited.

**Methods:**

A retrospective, record-based case-control study was conducted at the Kuska Wiñarisun Community Mental Health Center in Ayacucho, Peru, during 2022. The study included 122 adults with major depressive disorder: 61 cases with a documented history of suicide attempt and 61 controls without such history. Sociodemographic, clinical, psychosocial, and familial variables were extracted from clinical records using a structured data-collection form. Bivariate comparisons and logistic regression models were used to estimate crude and adjusted odds ratios (ORs) with 95% confidence intervals (CIs).

**Results:**

Among cases, 100 documented suicide attempts were recorded, with a mean of 1.64 ± 0.67 attempts per patient. In minimally adjusted exposure-specific models, history of suicide attempt was associated with alcohol dependence (adjusted OR = 3.21; 95% CI: 1.01–10.25), borderline personality disorder (adjusted OR = 2.64; 95% CI: 1.25–5.58), history of traumatic exposure or abuse (adjusted OR = 7.38; 95% CI: 1.54–35.31), and family history of suicide attempt (adjusted OR = 3.71; 95% CI: 1.52–9.05).

**Conclusion:**

These findings may inform trauma-informed, family-sensitive, and culturally responsive suicide-risk assessment for patients with major depressive disorder in underserved Andean community mental health settings.

## 1 Introduction

Suicide remains one of the most consequential outcomes in mental health: the Global Burden of Disease 2021 analysis estimated 746,000 deaths from suicide worldwide in 2021, including 519,000 among males and 227,000 among females (1). This burden occurs in parallel with the expanding global impact of major depressive disorder (MDD), for which the COVID-19 pandemic was associated with an estimated 53.2 million additional cases worldwide, equivalent to a 27.6% increase in prevalence (2). Among people with MDD, suicidality is substantially more frequent than in non-MDD populations; a systematic review and meta-analysis found higher odds of lifetime suicide attempts (OR = 3.45), past-year suicide attempts (OR = 7.34), and lifetime suicide plans (OR = 9.51) among patients with MDD compared with non-MDD controls (3). Population-based evidence also indicates that suicidal behavior identifies a clinically severe subgroup of MDD: in a Swedish cohort of 158,169 MDD episodes, 1.4% included recorded suicidal behavior and this group had higher all-cause mortality than matched MDD episodes without suicidal behavior (HR = 2.62) (4). Consistent with this clinical gradient, a systematic review of psychological autopsy studies found that any mental disorder (OR = 13.1) and history of self-harm (OR = 10.1) were among the strongest factors associated with suicide mortality in adults (5).

Within MDD, the risk of suicide attempt is not explained by depressive diagnosis alone, but by the accumulation of clinical, psychiatric, and psychosocial vulnerabilities. In a retrospective study of 1,091 patients with MDD, female sex, history of major mental trauma, impulsivity, family history of suicide, and depression severity were independently associated with suicidal behavior (6). Among outpatients with treatment-resistant depression, one-third had a history of suicide attempt, and substance use, psychiatric comorbidity, and higher Hamilton Depression Rating Scale scores were associated with suicide attempts (7). Borderline personality features are particularly relevant because a comparative study of patients with major depressive episodes reported lifetime suicide attempts in 16% of patients with MDD, 30% of those with bipolar depression, and 60% of those with comorbid borderline personality disorder (8). Broader evidence from personality-disorder research also supports the role of interpersonal instability, comorbid substance use, and childhood trauma as recurrent correlates of suicide-related outcomes (9). Somatic comorbidity may further compound vulnerability, as a meta-analysis of chronic pain populations reported lifetime suicidal ideation of 28.9% and lifetime suicide attempts of 10.8% across mixed chronic pain samples (10). Similarly, people living with HIV show a clinically important burden of suicidality, with pooled prevalence estimates of suicidal ideation, suicide attempts, and suicide deaths of 22.3%, 9.6%, and 1.7%, respectively, in a systematic review and meta-regression (11). Sexual minority status is another relevant dimension, as a global meta-analysis among men who have sex with men estimated pooled prevalences of suicidal ideation and suicide attempts of 21% and 12%, respectively (12).

In Peru, the need for locally grounded evidence is especially important because national and Andean mental health contexts differ from the settings where most suicide research has been produced. A national survey analysis of Peruvian adults found a 6.8% prevalence of depressive symptoms and reported that alcohol abuse was associated with higher odds of depressive symptoms (aOR = 1.88), underscoring the overlap between depression and modifiable behavioral risks (13). In rural Andean populations of Latin America during the COVID-19 pandemic, a systematic review found average symptom frequencies of 45.1% for anxiety, 27.6% for depression, and 33.1% for stress, while noting that suicide-related outcomes could not be robustly estimated because available studies were limited (14). Peru has expanded community-based mental health care through Community Mental Health Centers, and a time-series analysis of 58 centers reported 988,456 unique users between March 2019 and October 2021, with 39.4% classified as having common mental health problems and 7.4% as having severe mental health problems (15). However, professionals working with individuals at suicide risk in Peru have reported gaps in specialized training and treatment resources, suggesting that clinical risk identification remains a practical challenge in community settings (16). Implementation research on Peruvian Community Mental Health Centers has also shown that continuity-of-care programs are acceptable to users and providers, but depend on sustained coordination, trained teams, and operational support (17). More recent Peruvian evidence among LGBTQ+ youth found high levels of suicide-related outcomes and associations with depressive symptoms, anxiety symptoms, unmet mental healthcare needs, and lower perceived acceptance, reinforcing the importance of studying socially patterned risk in the Peruvian context (18).

Despite this evidence, few studies have examined factors associated with history of suicide attempt among patients with MDD receiving care in community mental health services in the Peruvian Andes. Most available studies come from high-income countries, hospital-based samples, general population surveys, or non-Andean settings, which limits their applicability to community mental health centers serving socially and clinically vulnerable populations.

Therefore, this case-control study aimed to identify sociodemographic, clinical, and psychosocial factors associated with history of suicide attempt among patients with major depressive disorder treated at a Community Mental Health Center in the Peruvian Andes.

## 2 Materials and methods

### 2.1 Study design and setting

We conducted a retrospective, record-based case–control study at the Kuska Wiñarisun Community Mental Health Center, located in Jesús Nazareno, Ayacucho, Peru, during 2022. The center provides community-based mental health care for a predominantly Andean population and receives patients with depressive disorders referred or identified through local mental health services.

### 2.2 Participants

The study included adult patients with a clinical diagnosis of major depressive disorder who received care at the Kuska Wiñarisun Community Mental Health Center during 2022. Eligibility required at least two documented clinical consultations to permit verification of key clinical, psychosocial, and suicide-attempt-related information across follow-up records rather than relying solely on the initial consultation. This criterion was applied uniformly to cases and controls.

Cases were patients with major depressive disorder and at least one documented suicide attempt in the available clinical record. Controls were patients with major depressive disorder who met the same eligibility criteria but had no documented history of suicide attempt. Controls were selected from the same clinical source population and calendar year as cases, and no matching procedures were applied.

Medical records were excluded if they corresponded to patients younger than 18 years, patients without a diagnosis of major depressive disorder, patients whose main psychiatric diagnosis was a condition other than major depressive disorder, or records that were illegible or contained insufficient information to determine case–control status or the main study variables.

The final analytic sample comprised 122 patients: 61 cases and 61 controls. The 1:1 case-to-control ratio reflected the number of eligible control records available within the retrospective dataset authorized for this study. Expanding the control group would have required reviewing additional medical records beyond the scope of the approved protocol.

### 2.3 Outcomes

The primary outcome was a documented history of suicide attempt among patients with major depressive disorder. It was analyzed as a binary variable: at least one documented suicide attempt versus no documented suicide attempt in the available clinical record.

Sociodemographic, clinical, psychosocial, and familial variables were abstracted from clinical records using a structured data-collection form developed for this study. These variables included marital status, religious affiliation, alcohol dependence, borderline personality disorder, schizophrenia, painful chronic medical conditions, HIV/AIDS, age group, sex, sexual minority status, parental loss, history of traumatic exposure or abuse, and family history of suicide attempt. Alcohol dependence was the only substance-related diagnosis consistently documented in the available clinical records and predefined in the data-collection form. Information on non-alcohol psychoactive substance use was not recorded with sufficient consistency or clinical detail to permit reliable analysis. For regression analyses, marital status was dichotomized as single versus all other marital-status categories. Alcohol dependence, borderline personality disorder, schizophrenia, painful chronic medical conditions, HIV/AIDS, sexual minority status, history of traumatic exposure or abuse, and family history of suicide attempt were coded as binary variables based on explicit documentation in the clinical records.

Among cases, suicide-attempt characteristics were also abstracted, including the number of documented attempts and the timing of the most recent documented attempt. The number of attempts was summarized as a continuous variable using mean, standard deviation, median, and range, and was also categorized as one, two, or three attempts. The timing of the most recent documented attempt was categorized as occurring within the previous month or between one and two months before evaluation.

#### 2.3.1 Clinical record abstraction and bias control

To minimize information bias, data were collected retrospectively using a standardized abstraction form with predefined operational definitions. For eligible records, key exposures and suicide-attempt history were verified across the available consultation and follow-up notes. Before data extraction, the form underwent content and face validation by an expert panel with experience in psychiatry, epidemiology, and biostatistics. The experts reviewed the relevance, clarity, and consistency of all items, and the final version was approved by consensus before use.

Alcohol dependence and borderline personality disorder were abstracted as clinical diagnoses when they were explicitly documented in the medical record. History of traumatic exposure or abuse and family history of suicide attempt were abstracted from the clinical history and follow-up notes. For all variables, the study retained the information as recorded during routine clinical care, and a variable was coded as present only when it was explicitly documented in the available records. The research team did not independently reassess participants or retrospectively assign diagnoses. Moreover, the use of standardized diagnostic interviews or research instruments could not be verified consistently across all clinical records. The expert review described above assessed the abstraction form and its operational definitions but did not constitute independent validation of the diagnoses or clinical histories documented in the records. When files were illegible or contained insufficient information to determine case-control status or the main study variables, they were excluded from the analysis. Variables with very low prevalence or sparse cells, including schizophrenia, HIV/AIDS, and sexual minority status, were summarized descriptively but were not included in adjusted regression models because of potential model instability. To protect confidentiality, all extracted data were coded before analysis, and direct personal identifiers were excluded from the analytical database.

### 2.4 Statistical analysis

All analyses were performed using Stata version 19.5. Descriptive statistics were used to summarize the study population. Categorical variables were reported as absolute frequencies and percentages. Continuous variables, specifically the number of documented suicide attempts among cases, were summarized using the mean and standard deviation, as well as the median and range.

Bivariate comparisons between cases and controls were performed for sociodemographic, clinical, psychosocial, and familial characteristics. Pearson’s chi-square test was used for categorical variables when assumptions were met, and Fisher’s exact test was applied for variables with sparse cells or expected frequencies below five. Two-sided p-values below 0.05 were considered statistically significant.

Crude odds ratios (ORs) with 95% confidence intervals (CIs) were estimated using unadjusted logistic regression models. For adjusted analyses, separate minimally adjusted exposure-specific logistic regression models were fitted. Age group and sex were selected *a priori* as core demographic covariates because they could plausibly confound the associations between the exposures of interest and history of suicide attempt. Given the modest sample size and the sparse distribution of several exposures, particularly alcohol dependence and traumatic exposure or abuse, fitting a single model containing all candidate variables could have produced overfitting, unstable coefficients, and imprecise estimates. Therefore, each adjusted model included one primary exposure of interest together with age group and sex. When sex was evaluated as the exposure, the model was adjusted only for age group. The exposures evaluated were single marital status, religious affiliation, alcohol dependence, borderline personality disorder, female sex, history of traumatic exposure or abuse, and family history of suicide attempt. Variables with sparse cells were not included in adjusted analyses because of potential model instability. No fully mutually adjusted model containing all principal exposures was fitted. Consequently, the adjusted ORs should be interpreted as minimally adjusted exposure-specific estimates and not as evidence that each association was independent of the other clinical, psychiatric, psychosocial, or familial variables.

Because marital status was presented using its original categories in **Table 1** but dichotomized for regression analysis, the p-value in **Table 1** represents the global comparison across all marital-status categories, whereas the crude p-value in **Table 2** represents the binary comparison of single versus other marital status. Results are presented as crude and adjusted ORs with 95% CIs. All analyses were conducted in accordance with the Strengthening the Reporting of Observational Studies in Epidemiology recommendations.

**Table 1:** Sociodemographic, clinical, and psychosocial characteristics according to history of suicide attempt among patients with major depressive disorder.

Variable	Total n = 122 (%)	Cases with history of suicide attempt n = 61 (%)	Controls without history of suicide attempt n = 61 (%)	P-value
Marital status				
Single	79 (64.75)	44 (72.13)	35 (57.38)	0.381
Cohabiting	24 (19.67)	9 (14.75)	15 (24.59)	
Married	12 (9.84)	4 (6.56)	8 (13.11)	
Divorced	3 (2.46)	2 (3.28)	1 (1.64)	
Widowed	4 (3.28)	2 (3.28)	2 (3.28)	
Religious affiliation				
Yes	61 (50.00)	30 (49.18)	31 (50.82)	0.856
No	61 (50.00)	31 (50.82)	30 (49.18)	
Alcohol dependence				
Yes	16 (13.11)	12 (19.67)	4 (6.56)	0.032
No	106 (86.89)	49 (80.33)	57 (93.44)	
Borderline personality disorder				
Yes	56 (45.90)	35 (57.38)	21 (34.43)	0.011
No	66 (54.10)	26 (42.62)	40 (65.57)	
Schizophrenia				
Yes	8 (6.56)	5 (8.20)	3 (4.92)	0.717*
No	114 (93.44)	56 (91.80)	58 (95.08)	
Painful chronic medical condition				
Yes	17 (13.93)	9 (14.75)	8 (13.11)	0.794
No	105 (86.07)	52 (85.25)	53 (86.89)	
HIV/AIDS				
Yes	2 (1.64)	1 (1.64)	1 (1.64)	1.000*
No	120 (98.36)	60 (98.36)	60 (98.36)	
Age group				
Young adult (18–29 years)	71 (58.20)	41 (67.21)	30 (49.18)	0.127
Adult (30–59 years)	48 (39.34)	19 (31.15)	29 (47.54)	
Older adult (≥60 years)	3 (2.46)	1 (1.64)	2 (3.28)	
Sex				
Female	96 (78.69)	48 (78.69)	48 (78.69)	1.000
Male	26 (21.31)	13 (21.31)	13 (21.31)	
Sexual minority status				
Yes	5 (4.10)	3 (4.92)	2 (3.28)	1.000*
No	117 (95.90)	58 (95.08)	59 (96.72)	
Parental loss				
≤1 year	5 (4.10)	2 (3.28)	3 (4.92)	0.269
>1 year	11 (9.02)	8 (13.11)	3 (4.92)	
No	106 (86.89)	51 (83.61)	55 (90.16)	
History of traumatic exposure or abuse				
Yes	107 (87.70)	59 (96.72)	48 (78.69)	0.003
No	15 (12.30)	2 (3.28)	13 (21.31)	
Family history of suicide attempt				
Yes	30 (24.59)	22 (36.07)	8 (13.11)	0.003
No	92 (75.41)	39 (63.93)	53 (86.89)	

Values are presented as n (%), with percentages calculated within columns. *Fisher’s exact test. Abbreviations: HIV, human immunodeficiency virus; AIDS, acquired immunodeficiency syndrome.

**Table 2:** Logistic regression models for factors associated with history of suicide attempt among patients with major depressive disorder.

Variable	Crude OR	95% CI	P-value	Adjusted OR	95% CI	P-value
Single marital status	1.92	0.90–4.09	0.088	1.85	0.84–4.07	0.125
Religious affiliation	0.94	0.46–1.91	0.856	0.91	0.43–1.92	0.805
Alcohol dependence	3.49	1.06–11.52	0.032	3.21	1.01–10.25	0.048
Borderline personality disorder	2.56	1.22–5.37	0.011	2.64	1.25–5.58	0.011
Female sex	1.00	0.42–2.38	1.000	1.00	0.41–2.43	1.000
History of traumatic exposure or abuse	7.99	1.67–38.23	0.003	7.38	1.54–35.31	0.012
Family history of suicide attempt	3.74	1.50–9.36	0.003	3.71	1.52–9.05	0.004

Reference categories were other marital status, no religious affiliation, no alcohol dependence, no borderline personality disorder, male sex, no history of traumatic exposure or abuse, and no family history of suicide attempt. Adjusted ORs were obtained from separate minimally adjusted exposure-specific models and were not mutually adjusted for the other exposures listed in the table. Abbreviations: OR, odds ratio; CI, confidence interval.

## 3 Results

The study included 122 patients with major depressive disorder, including 61 cases with a documented history of suicide attempt and 61 controls without such history.

**Table 1** presents the sociodemographic, clinical, and psychosocial characteristics according to history of suicide attempt. Alcohol dependence was more frequent among cases than controls (19.7% vs. 6.6%; p = 0.032). Comorbidity with borderline personality disorder was also more frequent among cases than controls (57.4% vs. 34.4%; p = 0.011). History of traumatic exposure or abuse was highly frequent in the overall sample and was more common among cases than controls (96.7% vs. 78.7%; p = 0.003). Similarly, family history of suicide attempt was more frequent among cases than controls (36.1% vs. 13.1%; p = 0.003). Religious affiliation, schizophrenia, painful chronic medical conditions, HIV/AIDS, age group, sex, sexual minority status, and parental loss did not differ significantly between cases and controls.

Among cases with a documented history of suicide attempt, the mean number of documented suicide attempts was 1.64 ± 0.67, with a median of 2 attempts and a range from 1 to 3 attempts (**Table 3**). Most cases had one or two documented attempts. Regarding timing, 15 cases (24.6%) had their most recent documented attempt within the previous month, while 46 cases (75.4%) had their most recent documented attempt between one and two months before evaluation.

**Table 3:** Frequency and timing of documented suicide attempts among cases with major depressive disorder.

Documented suicide attempts among cases	n = 61	%
Number of attempts, mean ± SD	1.64 ± 0.67	—
Number of attempts, median (range)	2 (1-3)	—
Total number of documented attempts among cases	100	—
1 attempt	29	47.54
2 attempts	25	40.98
3 attempts	7	11.48
Timing of most recent documented attempt		
Within the previous month	15	24.59
Between one and two months before evaluation	46	75.41

were calculated among cases with a documented history of suicide attempt (n = 61).

**Table 2** summarizes the logistic regression models for factors associated with history of suicide attempt. In minimally adjusted exposure-specific logistic regression models, alcohol dependence was associated with history of suicide attempt after adjustment for age group and sex (adjusted OR = 3.21; 95% CI: 1.01–10.25; p = 0.048). Comorbidity with borderline personality disorder was also associated with higher odds of history of suicide attempt (adjusted OR = 2.64; 95% CI: 1.25–5.58; p = 0.011). History of traumatic exposure or abuse was associated with case status (adjusted OR = 7.38; 95% CI: 1.54–35.31; p = 0.012). However, the wide confidence interval indicates substantial uncertainty regarding the magnitude of this association. Family history of suicide attempt was also associated with history of suicide attempt after adjustment (adjusted OR = 3.71; 95% CI: 1.52–9.05; p = 0.004). Single marital status, religious affiliation, and female sex were not statistically significant in adjusted models. Because the models were fitted separately for each exposure, these estimates were not mutually adjusted for the other clinical, psychiatric, psychosocial, or familial variables listed in **Table 2**.

## 4 Discussion

In this retrospective case-control study of patients with major depressive disorder treated at a community mental health center in the Peruvian Andes, a documented history of suicide attempt was associated with alcohol dependence, comorbid borderline personality disorder, a history of traumatic exposure or abuse, and a family history of suicide attempt. These findings indicate that suicide-risk assessment in this setting may benefit from considering substance-related diagnoses, psychiatric comorbidity, trauma-related history, and familial vulnerability alongside major depressive disorder. However, the exposures and outcome were ascertained retrospectively from clinical records, and their temporal sequence could not be established. Accordingly, the findings identify associated clinical and psychosocial characteristics but do not demonstrate that these variables caused or predicted suicide attempts.

First, alcohol dependence emerged as a relevant clinical correlate of history of suicide attempt in our study. This finding is consistent with Holma et al. (19), who reported that alcohol use disorder was present in 24.5% of psychiatric patients with major depressive disorder and was associated with worse longitudinal outcomes, including more suicidal behavior during follow-up. Similarly, Richards et al. (20) found that, among adults receiving outpatient mental health care, high-level alcohol use was associated with a higher 90-day risk of suicide attempt (OR = 1.77; 95% CI, 1.22–2.57), while daily or almost daily heavy drinking showed an even stronger association (OR = 2.33; 95% CI, 1.38–3.93). Olsson et al. (21) also reported subsequent fatal or nonfatal suicidal behavior in 26% of patients with alcohol use disorder versus 18% of those without it, with the association remaining significant after adjustment (OR = 1.49; 95% CI, 1.04–2.15). The stronger association observed in our sample may reflect the clinical vulnerability of depressed patients treated in a community mental health setting, where alcohol dependence may coexist with psychosocial adversity and limited continuity of care. These findings support routine alcohol-use assessment as a key component of suicide-risk formulation in patients with major depressive disorder.

Second, borderline personality disorder was significantly associated with history of suicide attempt in our sample. This result agrees with Söderholm et al. (8), who found marked differences in lifetime suicide-attempt prevalence across mood-disorder subgroups, with attempts reported in 16% of patients with major depressive disorder, 30% of those with bipolar disorder, and 60% of those with borderline personality disorder; among patients with comorbid bipolar disorder and borderline personality disorder, the prevalence exceeded 90%. The same study reported that the severity of borderline personality disorder features was independently associated with suicide attempts both lifetime and during the current major depressive episode. Likewise, Yen et al. (22) found that borderline personality disorder was the strongest disorder associated with prospectively observed suicide attempts over 10 years of follow-up (OR = 4.18; 95% CI, 2.68–6.52). Grilo and Udo (23) also reported higher lifetime suicide-attempt prevalence among adults meeting criteria for borderline personality disorder than among those who did not (22.7% vs. 2.9%), with an adjusted OR of 2.18. Our findings therefore reinforce the need to assess personality disorder comorbidity when evaluating suicide risk in depressed patients.

Third, history of traumatic exposure or abuse was associated with history of suicide attempt in our study. This finding is consistent with Liu et al. (6), who reported that major mental trauma history was independently associated with suicidal behavior among inpatients with major depressive disorder, with ORs of 1.548 and 1.571 across adjusted models. It also aligns with the meta-analysis by Angelakis et al. (24), which pooled data from 68 studies and found that complex childhood abuse was associated with a markedly increased risk of suicide attempts in adulthood (OR = 5.18; 95% CI, 2.52–10.63). In a more recent prospective clinical cohort, Demesmaeker et al. (25) found that PTSD was associated with suicide reattempts within six months (OR = 1.71; 95% CI, 1.14–2.55), and that PTSD comorbid with substance use disorder showed an even stronger association (OR = 2.91; 95% CI, 1.28–6.62). In our study, the estimate for traumatic exposure or abuse had a wide confidence interval, reflecting substantial imprecision, likely related to the modest sample size and the sparse distribution of participants without documented traumatic exposure or abuse. Therefore, the point estimate should be interpreted cautiously and should not be used to infer the precise magnitude or relative clinical importance of this association.

Fourth, family history of suicide attempt was also associated with history of suicide attempt after adjustment. This finding is consistent with Liu et al. (6), who reported that family history of suicide was independently associated with suicidal behavior among patients with major depressive disorder (OR = 1.820; 95% CI, 1.044–3.174). Although their exposure was family history of suicide rather than family history of suicide attempt, the direction of association supports the familial clustering of suicidal behavior. Older evidence from mood-disorder samples also showed that suicidal behavior was more frequent among first-degree relatives of mood-disorder patients with a suicide attempt than among relatives of mood-disorder patients without such history (26), suggesting that familial vulnerability may operate through genetic liability, shared adversity, psychiatric comorbidity, and exposure to suicidal behavior. The stronger association in our study may reflect the use of a clinically direct variable family history of suicide attempt in a case-control sample enriched for suicidal behavior. This finding supports incorporating family history into routine clinical interviews, not as a deterministic marker, but as part of a broader psychosocial and psychiatric risk formulation.

Finally, several variables were not significantly associated with history of suicide attempt in the adjusted models, including single marital status, religious affiliation, and female sex. The absence of an association with female sex is understandable because cases and controls had identical sex distribution in our sample. This finding is consistent with Jiang et al. (27), who reported no significant gender difference in suicide-attempt prevalence among young first-episode, drug-naive patients with major depressive disorder and anxiety symptoms, with attempts documented in 23.80% of males and 26.12% of females. Zhu et al. (28) similarly reported no sex difference in suicide-attempt prevalence among first-episode, drug-naive patients with major depressive disorder. However, our finding differs from Liu et al. (6), who found female gender to be associated with suicidal behavior in hospitalized patients with major depressive disorder (OR = 1.492; 95% CI, 1.062–2.097). Regarding marital status and religion, the literature is mixed: some studies suggest higher risk among unmarried individuals, whereas findings for religious affiliation vary across cultural and clinical contexts. In our study, these broad categories may have been insufficient proxies for social support, family functioning, interpersonal conflict, or religious coping. Future research should include more granular psychosocial measures to better characterize suicide-risk profiles in underserved Andean settings.

This study contributes to the limited evidence on suicide-related clinical profiles among patients with major depressive disorder receiving care in community mental health services in the Peruvian Andes. Its main scientific contribution is the identification of a pattern of associated clinical, psychiatric, trauma-related, and familial characteristics in an underserved Andean setting. These findings are particularly relevant because most available evidence on suicide attempts in major depressive disorder comes from high-income countries, hospital-based samples, or non-Andean populations, limiting its direct applicability to community-based services in Latin America. Although the results cannot be generalized to all patients with major depressive disorder in Peru, they may be transferable to similar community mental health settings serving socially vulnerable populations with limited access to specialized psychiatric care. Accordingly, these findings may inform culturally responsive, trauma-informed, and family-sensitive assessment strategies in decentralized mental health systems.

This study has several strengths. First, cases and controls were selected from the same community mental health center and during the same calendar year, which reduced differences related to the care setting and access to services. Second, eligibility required at least two documented clinical consultations, which improved verification of suicide-attempt history and relevant exposures across follow-up records. Third, the study examined clinical, psychiatric, psychosocial, and familial factors using a structured data-collection form that underwent expert review.

However, several limitations should be acknowledged. The retrospective design relied on the completeness and accuracy of clinical records, which may have resulted in information bias or underdocumentation of sensitive variables. In addition, alcohol dependence, borderline personality disorder, traumatic exposure or abuse, and family history of suicide attempt were based on routine clinical documentation rather than uniform standardized research assessments. These exposures may therefore have been underdocumented or misclassified, and the direction and magnitude of any resulting bias cannot be determined. Although the requirement for at least two documented clinical consultations was applied equally to cases and controls, it may have introduced selection bias by preferentially including patients with greater continuity of care or more complex clinical needs while excluding those who attended only once. Consequently, the study sample may not fully represent all patients with major depressive disorder receiving care at the center, particularly those with limited engagement or early loss to follow-up, which may restrict the external validity of the findings. In addition, non-alcohol substance use could not be analyzed because it was not systematically or consistently documented in the available records, and its potential association with history of suicide attempt therefore remains unexamined.

The modest sample size limited statistical precision, and several adjusted estimates had wide confidence intervals. This was particularly evident for traumatic exposure or abuse, for which the adjusted estimate had a 95% confidence interval ranging from 1.54 to 35.31. Accordingly, the point estimates should be interpreted cautiously and should not be used to rank the magnitude or clinical relevance of the associated factors. Although including more than one control per case could have improved the precision of the estimates and the statistical power of the study, expanding the control group was not feasible within the approved retrospective dataset. The modest sample size and sparse distribution of some exposures also precluded fitting a stable fully mutually adjusted model containing all principal variables. Consequently, the exposure-specific estimates may remain confounded by the other clinical, psychiatric, psychosocial, or familial characteristics evaluated, and the independence of the reported associations cannot be established. Moreover, the case-control design precludes causal inference and does not establish the temporal sequence between the exposures and suicide-attempt history. Finally, the study was conducted at a single community mental health center; therefore, the findings should be interpreted cautiously when applied to other regions or healthcare settings.

In conclusion, among patients with major depressive disorder treated at a community mental health center in the Peruvian Andes, a documented history of suicide attempt was associated with alcohol dependence, borderline personality disorder, traumatic exposure or abuse, and family history of suicide attempt. These findings identify clinical, psychiatric, trauma-related, and familial characteristics that may be relevant to suicide-risk assessment in similar community mental health settings. Given the retrospective case-control design and record-based ascertainment of both exposures and outcome, these associations should not be interpreted as causal or predictive.

## Data Availability

The datasets analyzed for this study are not publicly available because they contain confidential clinical information. Requests to access the datasets should be directed to the corresponding author and will be considered in accordance with applicable institutional and ethical requirements.

## References

[1] GBD 2021 Suicide Collaborators . Global, regional, and national burden of suicide, 1990-2021: a systematic analysis for the Global Burden of Disease Study 2021. Lancet Public Health. (2025) 10:e189–202. doi: 10.1016/S2468-2667(25)00006-4 39986290 PMC11876099

[2] Santomauro DF Herrera AMM Shadid J Zheng P Ashbaugh C Pigott DM . Global prevalence and burden of depressive and anxiety disorders in 204 countries and territories in 2020 due to the COVID-19 pandemic. Lancet. (2021) 398:1700–12. doi: 10.1016/S0140-6736(21)02143-7 34634250 PMC8500697

[3] Cai H Xie XM Zhang Q Cui X Lin JX Sim K . Prevalence of suicidality in major depressive disorder: a systematic review and meta-analysis of comparative studies. Front Psychiatry. (2021) 12. doi: 10.3389/fpsyt.2021.690130 34603096 PMC8481605

[4] Lundberg J Cars T Lampa E Ekholm Selling K Leval A Gannedahl A . Determinants and outcomes of suicidal behavior among patients with major depressive disorder. JAMA Psychiatry. (2023) 80:1218–25. doi: 10.1001/jamapsychiatry.2023.2833 37585196 PMC10433143

[5] Favril L Yu R Uyar A Sharpe M Fazel S . Risk factors for suicide in adults: systematic review and meta-analysis of psychological autopsy studies. Evid Based Ment Health. (2022) 25:148–55. doi: 10.1136/ebmental-2022-300549 36162975 PMC9685708

[6] Liu C Pan W Zhu D Meng F Tian T Li L . Factors of suicidal behavior among inpatients with major depressive disorder: a retrospective case series. Front Psychiatry. (2022) 13. doi: 10.3389/fpsyt.2022.996402 36213915 PMC9537680

[7] Civardi SC Besana F Carnevale Miacca G Mazzoni F Arienti V Politi P . Risk factors for suicidal attempts in a sample of outpatients with treatment-resistant depression: an observational study. Front Psychiatry. (2024) 15. doi: 10.3389/fpsyt.2024.1371139 38585482 PMC10995380

[8] Söderholm JJ Socada JL Rosenström T Ekelund J Isometsä ET . Borderline personality disorder with depression confers significant risk of suicidal behavior in mood disorder patients—a comparative study. Front Psychiatry. (2020) 11. doi: 10.3389/fpsyt.2020.00290 32362847 PMC7181053

[9] McClelland H Cleare S O’Connor RC . Suicide risk in personality disorders: a systematic review. Curr Psychiatry Rep. (2023) 25:405–17. doi: 10.1007/s11920-023-01440-w 37642809 PMC10506938

[10] Kwon CY Lee B . Prevalence of suicidal behavior in patients with chronic pain: a systematic review and meta-analysis of observational studies. Front Psychol. (2023) 14. doi: 10.3389/fpsyg.2023.1217299 37842717 PMC10576560

[11] Tsai YT Padmalatha S Ku HC Wu YL Yu T Chen MH . Suicidality among people living with HIV from 2010 to 2021: a systematic review and a meta-regression. Psychosom Med. (2022) 84:924–39. doi: 10.1097/PSY.0000000000001127 36162070 PMC9553271

[12] Nouri E Moradi Y Moradi G . The global prevalence of suicidal ideation and suicide attempts among men who have sex with men: a systematic review and meta-analysis. Eur J Med Res. (2023) 28:361. doi: 10.1186/s40001-023-01338-6 37735701 PMC10514985

[13] Vasquez-Chavesta AZ Caira-Chuquineyra B Fernandez-Guzman D Llamo-Vilcherrez AP Barboza JJ Toro-Huamanchumo CJ . Health behaviors and depressive symptoms in Peruvian adults: a national survey analysis. J Affect Disord Rep. (2024) 16:100733. doi: 10.1016/j.jadr.2024.100733 38826717

[14] Moya-Salazar J Jaime-Quispe A Cañari B Moya-Espinoza JG Contreras-Pulache H . A systematic review of mental health in rural Andean populations in Latin America during the COVID-19 pandemic. Front Psychiatry. (2023) 14. doi: 10.3389/fpsyt.2023.1136328 37663592 PMC10470633

[15] Villarreal-Zegarra D Segovia-Bacilio P Paredes-Angeles R Vilela-Estrada AL Cavero V Diez-Canseco F . Provision of community mental health care before and during the COVID-19 pandemic: a time series analysis in Peru. Int J Soc Psychiatry. (2023) 69:1996–2006. doi: 10.1177/00207640231185026 37449754 PMC10350579

[16] Alonzo D Zapata Pratto DA . Mental health services for individuals at risk of suicide in Peru: attitudes and perspectives of mental health professionals. Int J Soc Psychiatry. (2021) 67:209–18. doi: 10.1177/0020764020946786 32847415

[17] Diez-Canseco F Rojas-Vargas J Toyama M Mendoza M Cavero V Maldonado H . Estudio cualitativo sobre la implementación del Programa de continuidad de cuidados y rehabilitación para personas con trastornos mentales graves en el Perú. Rev Panam Salud Pública. (2020) 44:e134. doi: 10.26633/RPSP.2020.134 33337443 PMC7737645

[18] Jauregui JC Reyes-Diaz M León-Morris F Nath R Taylor AB Konda KA . Understanding suicide risk among LGBTQ+ youth in Peru: findings from a nationwide mental health survey. Camb Prisms Glob Ment Health. (2026) 13:e50. doi: 10.1017/gmh.2026.10169 41929528 PMC13040301

[19] Holma M Holma I Isometsä E . Comorbid alcohol use disorder in psychiatric MDD patients: a five-year prospective study. J Affect Disord. (2020) 267:283–8. doi: 10.1016/j.jad.2020.02.024 32217228

[20] Richards JE Shortreed SM Simon GE Penfold RB Glass JE Ziebell R . Short-term risk of suicide attempt associated with patterns of patient-reported alcohol use determined by routine AUDIT-C among adults receiving mental healthcare. Gen Hosp Psychiatry. (2020) 62:79–86. doi: 10.1016/j.genhosppsych.2019.12.002 31874300 PMC7047881

[21] Olsson P Wiktorsson S Strömsten LMJ Salander Renberg E Runeson B Waern M . Clinical characteristics and 6-month follow-up of adults with and without alcohol use disorder who self-harm. Front Psychiatry. (2024) 15. doi: 10.3389/fpsyt.2024.1396855 39156607 PMC11327149

[22] Yen S Peters JR Nishar S Grilo CM Sanislow CA Shea MT . Association of borderline personality disorder criteria with suicide attempts: findings from the Collaborative Longitudinal Study of Personality Disorders over 10 years of follow-up. JAMA Psychiatry. (2021) 78:187–94. doi: 10.1001/jamapsychiatry.2020.3598 33206138 PMC7675214

[23] Grilo CM Udo T . Association of borderline personality disorder criteria with suicide attempts among US adults. JAMA Netw Open. (2021) 4:e219389. doi: 10.1001/jamanetworkopen.2021.9389 33974054 PMC8114135

[24] Angelakis I Gillespie EL Panagioti M . Childhood maltreatment and adult suicidality: a comprehensive systematic review with meta-analysis. Psychol Med. (2019) 49:1057–78. doi: 10.1017/S0033291718003823 30608046 PMC6498789

[25] Demesmaeker A Creupelandt C Leroy A Vaiva G D’Hondt F . Impact of posttraumatic stress disorder and comorbid psychiatric conditions on suicide reattempts. Eur J Psychotraumatol. (2025) 16:2461435. doi: 10.1080/20008066.2025.2461435 39936356 PMC11823379

[26] Baldessarini RJ Tondo L Pinna M Nuñez N Vázquez GH . Suicidal risk factors in major affective disorders. Br J Psychiatry J Ment Sci. (2019) 215:621–6. doi: 10.1192/bjp.2019.167 31292010

[27] Jiang D Lang X Wang D Zhang XY . Gender differences in risk factors for suicide attempts among young, first-episode and drug-naive major depressive disorder patients with anxiety symptoms. Front Psychiatry. (2024) 15. doi: 10.3389/fpsyt.2024.1424103 39176231 PMC11338873

[28] Zhu Y Zhang J Liu J Jia F Li Z Zhao X . Sex differences in suicide attempts: a cross-sectional study in patients with first-episode and drug-naïve major depression disorder. Depress Anxiety. (2024) 2024:5391546. doi: 10.1155/2024/5391546 40226653 PMC11918506

